# Construction and Surgical Training of Coronary Anastomosis on a Low-Cost Portable Simulator: Experience in a Peruvian Multicenter Study

**DOI:** 10.21470/1678-9741-2023-0479

**Published:** 2024-09-03

**Authors:** W Samir Cubas, Anna Paredes-Temoche, Wildor R. Dongo, Katherine E. Inga, Wilfredo Luna-Victoria, Enrique Velarde-Revilla

**Affiliations:** 1 Department of Thoracic and Cardiovascular Surgery, Cardiac Surgery Service, Edgardo Rebagliati Martins National Hospital, Lima, Peru; 2 Faculty of Medicine, Cayetano Heredia Peruvian University, Lima, Peru

**Keywords:** Operating Rooms, Surgeons, Clinical Competence, Internship and Residency, Curriculum, Thoracic Surgery

## Abstract

**Introduction:**

The operating room is no longer the ideal place for early surgica training of cardiothoracic surgery residents, forcing the search for simulation-based learning options. The study’s aim was the construction and surgicaltraining of coronary anastomosis in a portable, low-cost, homemade simulator.

**Methods:**

This is an observational, analytical, and multicenter study. The simulator was built with common materials and was evaluated with the Objective Structured Assessment ofTechnical Skills (or OSATS) Modified. All junior and senior residents from nine national cardiothoracic surgery centers were considered for 90 days. Operative skill acquisition and time in the creation of side-to-side (S-T-S), end-to-side (E-T-S), and end-to-end (E-T-E) coronary anastomoses were evaluated. All sessions were recorded and evaluated by a single senior cardiothoracic surgeon during two time periods.

**Results:**

One hundred and forty residents were assessed in 270 sessions. In junior residents, a significant improvement in final scores was identified in S-T-S (use of Castroviejo needle holder, needle angles, and needle transfer) (*P*<0.05). In seniors, a significant improvement was identified in S-T-S (graft orientation, appropriate spacing, use of forceps, angles, and needle transfer) anastomoses (*P*<0.05). A significant improvement in the final anastomosis time of senior residents over junior residents was identified in S-T-S (8.11 *vs.* 11.22 minutes), E-T-S (7.93 *vs.* 10.10 minutes), and E-T-E (6.56 *vs.* 9.68 minutes) (*P*=0.039).

**Conclusion:**

Our portable and low-cost coronary anastomosis simulator is effective in improving operative skills in cardiothoracic surgery residents; therefore, skills acquired through simulation-based training transfer have a positive impact on the surgical environment.

## INTRODUCTION

The development of the surgical learning curve among young cardiothoracic surgery residents has been progressively increasing, especially in frequent procedures such as coronary artery bypass grafting, where various skills related to coronary anastomoses are needed. However, the limited opportunities for training and acquisition of operative skills in the operating room, due to the classic axiom of patient safety and integrity above all, has been considered a paradigm that has allowed the development and innovation of operative simulation-based surgical education models^[1,2,3]^. This novel educational model has spread widely, and more and more specialist training programs are using simulation-based curricula, resulting in significant improvements in surgical performance and a great positive impact on the patient. Some studies describe figures showing a ~75% similarity or superiority (85.5% *vs.* 72.7%) in the acquisition of operative skills by simulation over classical learning techniques, especially procedures based on coronary anastomosis^[4,5,6,7]^. On the other hand, the implementation of these operative simulation programs, especially for common procedures such as coronary anastomosis, requires a large capital and economic investment of more than 150000 dollars per year, often becoming an obstacle for surgical training programs in developing countries with limited resources^[8,9,10,11]^. Therefore, our work aimed to propose the construction and surgical training of a portable and low-cost coronary anastomosis simulator, as the first multicenter experience among cardiothoracic surgery residents.

## METHODS

### Design and Sample Size

This is an observational, analytical, and multicenter study. The coronary vascular anastomosis simulator proposed by Cubas et al.^[12]^ was constructed and replicated, followed by the evaluation of training and acquisition of surgical skills with its use. The study sample consisted of all residents, junior (first, second, and third years) and senior (fourth and fifth years), from nine national cardiothoracic surgery centers during 2022. Assessment of the acquisition of operative skills and abilities was periodic and continuous (interdaily) for 90 days. All training sessions were recorded by video camera, timed, and evaluated at a second time point by a group of surgical mentors (senior surgeons). The sessions consisted of the creation of end-to-side (E-T-S), end-to-end (E-T-E), and side-to-side (S-T-S) coronary anastomoses, and finally, sessions that were not properly documented and evaluated during training were completely excluded from the study.

### Design, Construction, and Use of The Coronary Anastomosis Simulator

**Fig. 1:**
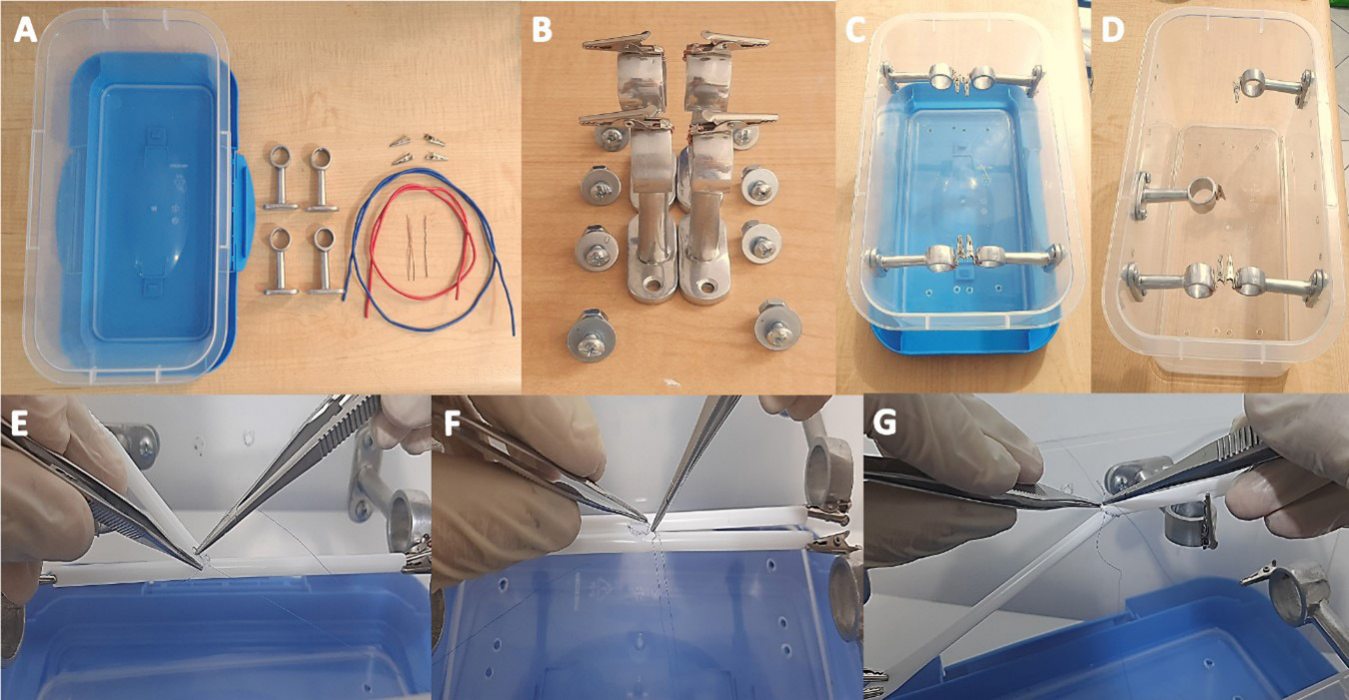
Materials, construction, and training of coronary anastomosis on the low-cost, portable anastomosis simulator.

The replication of this anastomosis simulator was based on the one proposed by Cubas et al.^[12]^, and the main principles were based on portability, ease of use, and high reproducibility (Figure 1). The materials used were inexpensive ($9.75) and included a plastic container with a lid, small crocodile hooks, fine galvanized wire, chrome-plated brackets for curtain installation, anchor bolts with nuts, and tools such as pliers and screwdrivers. The coronary anastomosis model was reproduced according to the previous prototype. The use of this simulator required thin plastic tubes as well as vascular surgical instruments (Castroviejo, vascular dissection forceps, scissors, and scalpel) and 6 and 7-0 polypropylene sutures. The thin plastic tubes were arranged parallel for S-T-S anastomoses and perpendicular for E-T-E and E-T-S anastomoses.

### Data Collection and Study Variables

The primary source of information was observation, and subsequently, the data were selected and sorted chronologically; finally, the data were collected using a double-entry data entry and checklist. The acquisition of operative skills was assessed according to the Objective Structured Assessment of Technical Skills (OSATS), and the components of this modified scale included graft orientation, bites, spacing, use of needle holder, use of forceps, needle angles, needle transfer, suture management, and tension and were individually scored as 1 (good), 2 (average), or 3 (poor) as observed by the surgical tutors^[12]^. Additionally, the variable of vascular anastomosis creation time was considered, and its value was counted in minutes. The scores of all described variables were grouped in three groups according to the type of coronary anastomosis (S-T-S, E-T-E, E-T-S), then in two groups according to the type of cardiothoracic surgery resident (junior *vs.* senior); and both modalities were evaluated in two 45-day time periods, first period (PP) *vs.* second period (SP).

### Statistical Analysis

Continuous data were expressed as mean ± standard deviation and analyzed with paired f-tests to compare scores obtained during FP *vs.* SP in the three types of anastomoses and taking into account the resident’s condition. It was not necessary to assess inter-rater reliability when scoring the anastomosis sessions, as all surgical mentors used the same surgical principles and the standardized OSATS modified score. Differences were considered significant at *P*-value < 0.05, and in all cases, data analysis was performed using STATA MP v16 statistical software for Windows 10.

### Ethical Aspects

The ethical evaluation and feasibility of this study was carried out by the Department of Thoracic and Cardiovascular Surgery and the Ethics Committee of the Hospital (RCEI-7/134_23), who reviewed and approved the protocol of this study. The confidentiality of the information and the principles of bioethics set out in the Declaration of Helsinki were respected.

## RESULTS

A total of 140 cardiothoracic surgery residents from nine academic training centers throughout the national territory were evaluated. The mean age was 29.16 years, and 69.28% of them were male. All were evaluated in 270 sessions and with an average of 4,904 minutes of operative performance per simulation. Of the residents, 55.71% were classified as juniors, and it was identified that in S-T-S anastomoses (FP *vs.* SP), there was a significant improvement in the use of Castroviejo needle holder (*P*=0.042), needle angles (*P*=0.036), needle transfer (*P*=0.029), and anastomosis time (25.39 min. *vs.* 11.22 min., *P*=0.037). In E-T-S anastomoses, a significant improvement was identified in graft orientation (*P*=0.049), appropriate spacing (*P*=0.031), use of Castroviejo needle holder (*P*=0.037), needle angles (*P*=0.041), suture management and tension (*P*=0.045), and anastomosis time (24.01 min. *vs.* 10.10 min., *P*=0.048). In E-T-E anastomoses, a significant improvement was identified in the use of a Castroviejo needle holder (*P*=0.045), needle transfer (*P*=0.018), and anastomosis time (19.55 min. *vs.* 9.58 min., *P*=0.041) (Table 1). Of the residents, 44.29% were classified as senior, and it was identified that in S-T-S anastomoses (FP *vs.* SP), there was a significant improvement in graft orientation (*P*=0.019), appropriate spacing (*P*=0.032), use of forceps (*P*=0.045), needle angles (*P*=0.009), needle transfer (*P*=0.043), and anastomosis time (19.21 min. *vs.* 8.11 min., *P*=0.021). In E-T-S anastomoses, a significant improvement was identified in graft orientation (*P*=0.041), appropriate bite (*P*=0.009), appropriate spacing (*P*=0.048), needle angles (*P*=0.048), needle transfer (*P*=0.012), suture management and tension (*P*=0.049), and anastomosis time (17.45 min. *vs.* 7.93 min., *P*=0.023). In E-T-E anastomoses, a significant improvement was identified in graft orientation (*P*=0.029), appropriate spacing (*P*=0.003), use of Castroviejo needle holder (*P*=0.009), use of forceps (*P*=0.026), needle angles (*P*=0.032), needle transfer (*P*=0.010), and anastomosis time (15.10 min. *vs.* 6.56 min., *P*=0.038) (Table 1).

**Table 1:** Modified OSATS scores in cardiothoracic surgery residents in the operative simulation of coronary anastomosis with the portable and low-cost simulator.

Variables		Coronary Anastomosis Simulator (Residents = 140/100%)								
Overall age ± SD (years)		29.16 ±3.78								
Male(n∕%)		97/69.28%								
		Anastomosis S-T-S			Anastomosis E-T-S			Anastomosis E-T-E		
**MODIFIED OSATS**	**Junior Resident (78 ∕ 55.71%)**	**FP**	**SP**	***P*-value**	**FP**	**SP**	***P*-value**	**FP**	**SP**	***P*-value**
	Graft orientation	2.56 ±0.32	2.01 ±0.43	0.091	2.87 ±0.11	1.91 ±0.18	**0.049**	2.24 ± 0.45	1.87 ± 0.32	0.073
	Appropriate bite	2.82 ±0.11	2.31 ±0.45	0.102	2.71 ±0.22	2.21 ±0.39	0.219	2.41 ± 0.24	2.10 ± 0.15	0.256
	Appropriate spacing	2.45 ± 0.43	2.10 + 0.18	0.096	2.55 ± 0.46	1.99 ± 0.25	**0.031**	2.65 ± 0.33	2.01 ±0.29	0.147
	Use of Castroviejo needle holder	2.67 ±0.21	1.45 ± 0.28	**0.042**	2.38 ± 0.34	1.29 ±0.14	**0.037**	2.55 ± 0.42	1.38 ± 0.29	**0.045**
	Use of forceps	2.21 ±0.56	1.72 ±0.21	0.158	2.18 ±0.46	1.81 ±0.25	0.192	2.34 ± 0.29	1.89 ±0.41	0.201
	Needle angles	2.76 ± 0.23	1.85 ± 0.22	**0.036**	2.79 ±0.19	1.61 ±0.33	**0.041**	2.49 ± 0.26	1.85 ± 0.28	0.055
	Needle transfer	2.85 ±0.12	1.62 ± 0.35	**0.029**	2.62 ±0.21	1.95 ± 0.26	0.051	2.69 ± 0.23	1.71 ±0.26	**0.018**
	Suture management and tension	2.34 ± 0.46	1.82 ±0.12	0.186	2.25 ±0.37	1.90 ± 0.29	0.079	2.46 ± 0.37	1.98 ±0.21	0.191
	**Overall Score**	2.58 ± 0.29	1.84 ±0.14	0.067	2.54 ±0.23	1.93 ± 0.34	**0.045**	2.47 ± 0.28	1.91 ±0.23	0.071
	**Anastomosis Time (min.)**	25.39	11.22	**0.037**	24.01	10.10	**0.048**	19.55	9.68	**0.041**
	**Senior Resident (62 ∕ 44.29%)**									
	Graft orientation	2.45 ±0.21	2.11 ±0.13	**0.019**	2.23 ±0.35	1.40 ± 0.23	**0.041**	2.12 ±0.30	1.67 ± 0.46	**0.029**
	Appropriate bite	2.71 ±0.38	2.19 ±0.40	0.143	2.67 ± 0.29	2.01 ±0.21	**0.009**	2.87 ± 0.49	2.98 ± 0.96	0.065
	Appropriate spacing	2.17 + 0.18	2.32 ± 0.23	**0.032**	2.11 ±0.78	1.34 ± 0.32	**0.048**	2.20 ±0.11	2.56 ± 0.56	**0.003**
	Use of Castroviejo needle holder	2.56 ±0.31	1.19 ±0.29	0.066	2.87 ±0.91	1.38 ± 0.56	0.090	2.98 ±0.19	1.33 ±0.21	**0.009**
	Use of forceps	2.27 ±0.32	1.10 ±0.33	**0.045**	2.56 ±0.31	1.76 ± 0.65	0.109	2.44 ± 0.67	1.67 ± 0.78	**0.026**
	Needle angles	2.66 ±0.11	1.56 ± 0.65	**0.009**	2.69 ± 0.45	1.89 ± 0.39	**0.048**	2.29 ± 0.78	1.89 ± 0.67	**0.032**
	Needle transfer	2.10 + 0.21	1.89 ± 0.32	**0.043**	2.45 ± 0.56	1.45 ±0.11	**0.012**	2.34 ± 0.78	1.32 ± 0.34	**0.010**
	Suture management and tension	2.21 ±0.34	1.76 ± 0.44	0.144	2.69 ± 0.30	1.67 ±0.18	**0.049**	2.78 ± 0.45	1.80 ± 0.98	0.104
	**Overall Score**	2.41 ±0.18	1.55 ±0.18	**0.046**	2.88 ± 0.49	1.32 ±0.91	0.079	2.43 ± 0.59	1.56 ± 0.78	0.198
	**Anastomosis Time (min.)**	19.21	8.11	**0.021**	17.45	7.93	**0.023**	15.10	6.56	**0.038**
	**End-Overall Average (Score/Anastomosis Time)**	**Junior Resident**				**Senior Resident**				**P-value**
	Anastomosis S-T-S	1.84 ±0.14/11.22 min.				1.55 ±0.18/8.11 min.				**0.039**
	Anastomosis E-T-S	1.93 ±0.34/10.10 min.				1.32 ±0.91/7.93 min.				
	Anastomosis E-T-E	1.91 ±0.23/9.68 min.				1.56 ± 0.78/6.56 min.				

E-T-E=end-to-end; E-T-S=end-to-side; FP=first period; OSATS=Objective Structured Assessment of Technical Skills; SD=standard deviation; SP=second period; S-T-S=side-to-side

**Fig. 2:**
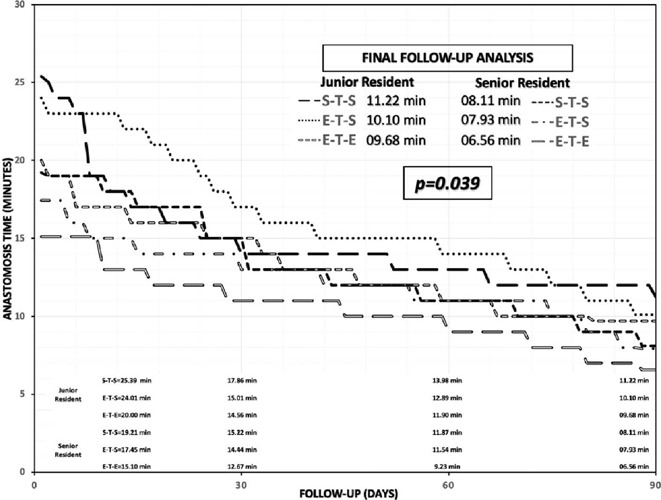
Follow-up on the acquisition of skills and abilities In the construction of coronary anastomosis with the simulator during 90 days In cardiothoracic surgery residents.

A significant improvement in the final anastomosis time of senior *vs.* junior residents was identified in S-T-S (8.11 min. *vs.* 11.22 min.), E-T-S (7.93 min. *vs.* 10.10 min.), and E-T-E (6.56 min. *vs.* 9.68 min.) anastomoses (*P*=0.039) (Figure 2).

## DISCUSSION

Traditional Halsteadian surgical education involves learning skills in an operating theatre in a progressive manner. Although this model has been widely used, multiple limitations may threaten the educational opportunities and learning of operative skills with this approach in the current era. In addition, with increasing public scrutiny of surgical outcomes and increasing complexity of surgical cases, surgeons are less inclined to engage residents, particularly when it comes to time, to complete a surgical procedure safely and efficiently™. These factors contribute to a high-stress environment that may be suboptimal for medical-surgical education. Thus, surgical simulation in the field of cardiovascular surgery allows for repetitive and safe training of resident skills. Particularly, coronary anastomosis is an important and basic skill set in cardiovascular surgery. In the present study, we found that junior and senior residents consistently benefited from the simulator, with a significant decrease in S-T-S, E-T-S, and E-T-E anastomosis time by at least 10 minutes in all cases. A recent study^[2]^ found that junior residents demonstrated a greater mean reduction in anastomosis time (6 minutes and 36 seconds) compared to senior residents (3 minutes and 6 seconds), and Whittaker et al.^[3]^ suggest that simulators should be used in the initial resident training when the learning curve for trainees is steepest.

Interestingly, in another paper, the data revealed a greater improvement in the senior resident group, speculating that this greater effect may be due to a more developed surgical skill set and a greater ability to benefit from repeated exposure to skills in this group^[1]^. In terms of motor skills, significant improvement was observed in the junior resident group in all types of anastomoses concerning the use of the Castroviejo needle holder, followed by improvement in needle manipulation in most types of anastomoses. While in the senior resident group, a vast improvement was observed in most motor skills: graft orientation, proper suture spacing, forceps use, and needle entry angle in all types of anastomoses.

A systematic review of coronary artery anastomosis performance showed that simulation was associated with significant improvement in all trainee scores in arteriotomy, graft orientation, depth, suture spacing, Castroviejo/needle holder use, forceps use, needle angles, needle transfer, suture handling, knots, manual mechanics, use of both hands, time economy, and anastomosis configuration^[4]^. In addition, Takahashi et al.^[5]^ demonstrated that formative feedback from mentors significantly improved motor skill components of coronary vascular anastomosis making, and if we add this expert-guided training to deliberate, independent practice by trainees, we obtain significantly higher scores on the OSATS scale^[8]^.

In other coronary anastomosis simulation models such as beating heart models, participating trainees showed improved ability concerning technical skills related to instrument handling®. Also, in the latter models using human cadavers, training in all types of anastomosis resulted in the recommendation of a score of at least 48 points on the OSATS before trainees could begin training with patients, progressively improving their score^[7]^.

Thus, according to our findings along with other studies, simulation was consistently associated with better learning outcomes in terms of anastomosis time. In addition, we highlight that senior residents benefit more from acquiring complex and meticulous motor skills. On the other hand, the results obtained translate into an improvement in both technique and time after the use of the simulator throughout the follow-up period. Regarding the end-overall average score, it was found that senior residents had a significant improvement in S-T-S anastomosis score (2.41 ± 0.18 to 1.55 ± 0.18; *P*=0.046) compared to junior residents (2.58 ± 0.29 to 1.84 ± 0.14; *P*=0.067). The latter may be contradictory to the usual findings, such as those found by Nesbitt and Anand et al.^[10]^ where junior residents tend to have a greater and significant improvement in score than senior residents^[9]^. This is often attributed to the greater amount of time junior residents may have to practice on the simulator compared to senior residents, who while more actively participating in surgery also have less time to practice on the simulator^[11,12,13]^.

This difference in findings found in our study population may be mainly due to the low patient load which is reflected in fewer surgical practice opportunities in both senior and junior residents. This is seen when comparing the overall average score of senior residents *vs.* junior residents at the beginning of the FP of our study, before the use of the simulator, and seeing that there is little difference between the two groups. This highlights the importance of the creation and use of simulators, such as ours, that allow continuous practice for surgical procedures.

Concerning the overall average time of anastomosis, a marked decrease could be observed as illustrated in the follow-up curve for all groups, with a significant improvement of senior residents over junior residents. This is contrasted with the results of the end-overall average score and confirms that senior residents had a greater and significant improvement compared to junior residents. This time improvement due to simulator training was also described by Tavlasoglu et al.^[14]^ whose results indicated a decrease in anastomosis time (13.65 ± 0.67 to 10.50 ± 1.10), a reduction in posterior wall damage (30% to 5%), and an increase in patency (80% to 95%) with acceptable statistical significance. Similarly, Fann et al.^[15]^, who designed a portable porcine coronary anastomosis simulator model, demonstrated a significant improvement in needle transfer (2.24 ± 0.49 to 1.58 ± 0.50) and suture management and tension (2.33 ± 0.62 to 1.58 ± 0.50). Because of these results and new teaching technique that allows them to practice without risk to patients, many simulation laboratories have been implemented in hospitals around the world^[16,17,18,19]^.

Although the transition from the simulator to surgical practice on real patients has not yet been extensively studied in our setting in the field of coronary anastomosis, it has been shown that after performing 30 anastomosis procedures on a simulator, the learning curve stabilizes, which is beneficial for the resident^[20,21,22]^. Time optimization through practice is beneficial not only for the residents but also for the patients when it comes to these procedures, as less time indicates an improvement in the surgeon’s skill, which means fewer complications. This was observed in a study evaluating 15 cardiothoracic surgeons, where the risk of mortality decreased significantly after four years of appointment, possibly due to the constant practice obtained^[23]^.

This type of simulation-guided training is now a tangible idea and is being promoted by organizations such as The Society of Thoracic Surgeons (or STS), The American College of Surgeons (or ACS), The Thoracic Surgery Residents Association (or TSRA), and others through the development of training and accreditation programs using simulators for residents and cardiothoracic surgeons^[24,25]^. Considering the national context, where most hospitals do not operate on coronary patients daily and the price of advanced simulators can be very high, our proposal for the construction and surgical training of a low-cost, portable coronary anastomosis simulator is the best option not only for residents but also for newly appointed surgeons.

## CONCLUSION

Simulator training should be incorporated into the curriculum of cardiovascular surgery programs and residents should be offered access to this modality to practice independently, as this increases opportunities for learning and motor skill development, seamlessly integrating technology for safe patient care.

## Abbreviations, Acronyms & Symbols

E-T-E: End-to-end
E-T-S: End-to-side
FP: First period
OSATS: Objective Structured Assessment of Technical Skills
SD: Standard deviation
SP: Second period
S-T-S: Side-to-side
